# Dr. Frank Abbott

**Published:** 1897-07

**Authors:** W. D. Tension, B. C. Nash, M. C. Gottschaldt

**Affiliations:** Committee.; Committee.; Committee.


### Obituary.

### RESOLUTIONS IN MEMORY OF DR. FRANK ABBOTT.

At a special meeting of the Alumni Association of the New York College of Dentistry, held April 19, 1897, the following preamble and resolutions were unanimously adopted :

"WHEREAS, In the wisdom of an all-wise Providence the Alumni Association of the New York College of Dentistry is called upon to mourn the loss by death of our late professor and dean, Frank Abbott, M. D.; therefore be it

"*Resolved*, That the death of our late professer and friend calls for an expression of esteem for one whose character was above reproach, whose undeviating justice and loving kindness to all with whom he came in contact left impressions that can not be effaced, and whose power for good, both in and outside his profession, was immeasurable. One of the brightest lights of our profession has been snuffed out, but its influence —pure and strong—cannot be extinguished while dentistry lasts.

"*Resolved*, That this premable and resolutions be placed in full upon the minutes, and that a copy suitably engrossed be presented to his bereaved family, for whom we feel the sincerest sympathy.

W. D. TENSION,
B. C. NASH,
M. C. GOTTSCHALDT,
Committee."

*Memorandum.*—Professor Frank Abbott, M. D., born September 5, 1836, died April 20, 1897. College of Dentistry, clinical instructor, 1866-8; established infirmary, 1867; superintendent of infirmary, 1867; professor and trustee, 1868; dean, 1860.

